# Comprehensive analysis of ferroptosis-related genes in immune infiltration and prognosis in multiple myeloma

**DOI:** 10.3389/fphar.2023.1203125

**Published:** 2023-08-07

**Authors:** Quanqiang Wang, Misheng Zhao, Tianyu Zhang, Bingxin Zhang, Ziwei Zheng, Zhili Lin, Shujuan Zhou, Dong Zheng, Zixing Chen, Sisi Zheng, Yu Zhang, Xuanru Lin, Rujiao Dong, Jingjing Chen, Honglan Qian, Xudong Hu, Yan Zhuang, Qianying Zhang, Songfu Jiang, Yongyong Ma

**Affiliations:** ^1^ Department of Hematology, The First Affiliated Hospital of Wenzhou Medical University, Wenzhou, Zhejiang, China; ^2^ Department of Clinical Laboratory, Wenzhou People’s Hospital, Wenzhou, China; ^3^ Department of Hepatobiliary Surgery, The Second Affiliated Hospital and Yuying Children’s Hospital of Wenzhou Medical University, Wenzhou, Zhejiang, China; ^4^ Key Laboratory of Intelligent Treatment and Life Support for Critical Diseases of Zhejiang Province, Wenzhou, Zhejiang, China; ^5^ Zhejiang Engineering Research Center for Hospital Emergency and Process Digitization, Wenzhou, Zhejiang, China

**Keywords:** multiple myeloma, ferroptosis, prognostic marker genes, overall survival, gene set enrichment analysis, drug sensitivity, immune analysis

## Abstract

**Background:** One particular type of cellular death that is known as ferroptosis is caused by the excessive lipid peroxidation. It is a regulated form of cell death that can affect the response of the tumor cells. Currently, it is not known if the presence of this condition can affect the prognosis of patients with multiple myeloma (MM).

**Methods:** In this study, we studied the expression differences and prognostic value of ferroptosis-related genes (FRGs) in MM, and established a ferroptosis risk scoring model. In order to improve the prediction accuracy and clinical applicability, a nomogram was also established. Through gene enrichment analysis, pathways closely related to high-risk groups were identified. We then explored the differences in risk stratification in drug sensitivity and immune patterns, and evaluated their value in prognostic prediction and treatment response. Lastly, we gathered MM cell lines and samples from patients to confirm the expression of marker FRGs using quantitative real-time PCR (qRT-PCR).

**Results:** The ability to predict the survival of MM patients is a challenging issue. Through the use of a risk model derived from ferroptosis, we were able to develop a more accurate prediction of the disease’s prognosis. They were then validated by a statistical analysis, which showed that the model is an independent factor in the prognosis of MM. Patients of high ferroptosis risk scores had a much worse chance of survival than those in the low-risk groups. The calibration and power of the nomogram were also strong. We noted that the link between the ferroptosis risk score and the clinical treatment was suggested by the FRG’s significant correlation with the immune checkpoint genes and the medication sensitivity. We validated the predictive model using qRT-PCR.

**Conclusion:** We demonstrated the association between FRGs and MM, and developed a new risk model for prognosis in MM patients. Our study sheds light on the potential clinical relevance of ferroptosis in MM and highlights its potential as a therapeutic target for patients with this disease.

## 1 Introduction

Uncontrolled plasma cell proliferation is the main cause of MM myeloma, which is a type of cancer that affects the bone marrow. Despite the advancements that have been made in treating this condition, the median overall survival rate for patients with this disease is still poor at only 5 years (Akhmetzyanova et al., 2020). Due to the existence of genetic abnormalities in MM patients, the importance of accurate risk stratification has been acknowledged. These abnormalities have been well-studied and are known to predict the prognosis of patients with this disease. (Abdallah et al., 2020) T (Palumbo et al., 2015; Bordini et al., 2020), T (Geeleher et al., 2014; Bordini et al., 2020), T (Bordini et al., 2020; Gonsalves et al., 2020), T (Rajkumar et al., 2014; Bordini et al., 2020), del (17/17p) and Nonhyperdiploid karyotype, karyotype del (Bordini et al., 2017) and gain (1q) were identified as important factors associated with poor prognosis. They can be used to form a predictive or prognostic score based on various clinical factors. Some of these include age, albumin, gender, and β2-microglobulin levels (Sonneveld et al., 2016). The Revised International Staging System (R-ISS) is also widely used to predict the prognosis of MM patients (Palumbo et al., 2015). The prognosis may vary significantly within each risk group indicated by the R-ISS, though. One study revealed that the R-ISS is more accurate than the International Staging System (ISS) when it comes to predicting a patient’s survival. However, it did not perform well when it came to assigning grades to the individuals (Cho et al., 2017). One study revealed that the R-ISS is more accurate than the International Staging System (ISS) when it comes to predicting a patient’s survival. However, it did not perform well when it came to assigning grades to the individuals. Several methodologies that could potentially enhance the precision of the R-ISS include analyzing blood clone plasma cells (Gonsalves et al., 2020), and imaging techniques such as positron emission tomography (Jung et al., 2019), and next-generation sequencing (Bolli et al., 2020). Unfortunately, these did not significantly increase R-ISS prediction accuracy.

Recently, people are starting to notice a new type of cell death known as ferroptosis, which is associated with various types of cancer. This mode of cell death is triggered by the imbalance between reactive oxygen species (ROS) and intracellular lipid peroxide. GPX4 and SLC7A11 are important regulators of ferroptosis and are highly expressed in MM cells. It was found that the immunosuppressant fingolimod (FTY720) could promote ferroptosis by reducing the mRNA and protein levels of GPX4 and SLC7A11 in U266 cells (Zhong et al., 2020). The process of lipid peroxide accumulation in cells requires the participation of iron ions, so ferroptosis is iron-dependent (Yan et al., 2021). In the study of MM model, plasma cells are very sensitive to excess iron. By producing antibodies, a large number of by-products such as H2O2 are synthesized, and finally ROS are stimulated by Fenton reaction (Tu and Weissman, 2004; Shimizu and Hendershot, 2009; Bordini et al., 2017). As a result, inducing iron overload may impede malignant plasma cell proliferation and improve the abilities bortezomib and carfilzomib to control disease development. Different MM cell lines were treated with high doses of ferrous ammonium citrate (FeAC), and untreated and other cell lines were used as controls for *in vitro* experiments. Iron caused cell death in MM cell lines, but not in control cells, and decreased proliferation was detected in all cell lines. Multiple myeloma cells exposed to iron undergo lipid oxidation and inhibit proteasome function, which means that the role of proteasome inhibitors will be enhanced (Campanella et al., 2013; Bordini et al., 2020). Our predictive model was developed using the FRGs public dataset and has demonstrated independent precision, and the R-ISS’ prognostic evaluation accuracy was further improved by incorporating it.

## 2 Materials and methods

### 2.1 Data acquisition

The gene expression profiles and clinicopathological information of three MM datasets GSE136337, GSE4204 and GSE24080 were obtained from the GEO database (http://www.ncbi.nlm.nih.gov/geo/), and the gene expression profiles were normalized between different arrays. Subsequently, we obtained 252 ferroptosis-related genes (FRGs) using the FerrDb database (www.zhounan.org/ferrdb).

### 2.2 Construction and validation of a ferroptosis risk score

The GSE136337 data set is moderate in size and has sufficient clinical data, including but not limited to R-ISS staging, ISS staging, genetic mutation information, etc., so we decided to use the GSE136337 data set as the final training data set for the generated ferroptosis risk score. In order to discover which FRGs were substantially linked with prognosis (*p* < 0.001), we utilized the GSE136337 data set and carried out a univariate Cox regression analysis. For the purpose of integrating the various data related to the study, such as the survival status, gene expression, and survival time, we made use of the “glmnet” R software package. Following that, we carried out a regression analysis utilizing the Lasso-Cox method in order to obtain the best possible model. Finally, the lambda value was determined to be 0.00387132108721305, and five FRGs were selected. We use GSE4204 and GSE24080 datasets to verify the model. The subjects were divided into two risk groups based on the median risk score of each data set. To assess the risk of ferroptosis, we used univariate and multivariate Cox regression analysis on the training set (GSE136337) and the validation set (GSE24080).

### 2.3 Constructing a predictive nomogram

The ROC curves were used to compare the prediction value of the R-ISS and the joint model with respect to the 3- and 5-year survival of patients. The AUC, which is a measure of the models’ accuracy, was used to analyze the differences between them.

When compared to the R-ISS, we discovered that the combined model had a significantly higher probability of correctly predicting the survival rate of multiple myeloma patients over the next three to five years. This suggests that the use of both the ferroptosis risk score and the R-ISS stage parameter can improve the accuracy of the joint model when it comes to identifying patients with this condition.

### 2.4 Gene set enrichment analysis

The Kyoto Encyclopaedia of Genes and Genomes (KEGG) pathway analysis is a widely used approach for pathway enrichment analysis. It helps to understand the biological function and interaction of genes and proteins by categorizing them into known pathways. Gene Set Enrichment Analysis (GSEA) is another widely used method for identifying differentially expressed genes and enriched pathways. It involves comparing the distribution of a pre-defined set of genes (e.g., a KEGG pathway) in two different groups of samples (high-risk vs. low-risk group) to determine whether the pathway is significantly enriched in one group compared to the other. In this study, the KEGG pathway was used to reveal the potential causes of ferroptosis risk score. GSEA was used to test the enrichment pathway in different ferroptosis risk score groups (GSEA v4.2.3 software, http://software.broadinstitute.org/gsea/login.jsp). Statistical significance was defined as *p* < 0.05, false discovery rate q < 0.25.

### 2.5 Drug sensitivity prediction

The “pRRophetic” (Geeleher et al., 2014) R package is a tool for predicting the half-maximal inhibitory concentration (IC50) of chemotherapeutic medicines using gene expression data. It uses a linear regression model that incorporates gene expression data from a training set of cell lines with known IC50 values for a given drug. The resulting model is then used to predict the IC50 values for new samples based on their gene expression profiles. This approach allows for the prediction of drug sensitivity or resistance in cancer patients and may be used to guide treatment decisions. We used the “pRRophetic” R package to test the drug sensitivity of the training set GSE136337 ferroptosis risk score high and low groups, and explored which drugs were different in the ferroptosis risk groups <0.05 was considered statistically significant.

### 2.6 Immune cell infiltration and immune microenvironment evaluation

The study utilized different algorithms for its analysis, such as the XCELL, TIDE, and CIBERSORT. The use of the XCELL algorithm can help determine the presence of various immune cells in the tumor microenvironment. This information can then be utilized to analyze the patterns of immune cell infiltration in the patients with MM of different risk group (Aran et al., 2017). The use of the CIBERSORT algorithm can also help identify the subsets of immune cells that are differentially expressed in patients with different risk levels. This method could provide a deeper analysis of the immune system’s response in patients (Chen et al., 2018). This can provide a deeper understanding of the immune response in different patient groups and can help identify potential targets for immunotherapy. The use of the TIDE algorithm can also help predict the response of the immune system to certain drugs in different patient groups. This method is useful in guiding the development of personalized treatment plans for patients with different types of cancer (Jiang et al., 2018). We also performed IPS scores on high- and low-risk groups to compare the different immune responses in each group.

### 2.7 Cell culture and reagents

The MM cell lines, namely H929, I9.2, U266, and RPMI 8226, were procured from Hunan Fenghui Biotechnology Co., Ltd. Gibco provided the cell culture and reagents. Short tandem repeat analysis was used to identify the cell lines, and they were routinely screened for *mycoplasma* contamination with Vazyme’s Myco-Blue *Mycoplasma* Detection Kit. The cells were grown in an incubator at 37 degrees Celsius and 5% carbon dioxide in a medium called RPMI 1640. The medium contained 10% fetal bovine serum, 100 international units per milliliter of penicillin, and 100 mg per milliliter of streptomycin.

### 2.8 Patient

31 MM patients were involved in the study at the Department of Clinical Hematology of the First Affiliated Hospital of Wenzhou Medical University, between January 2022 and November 2022. The International Myeloma Working Group (IMWG) criteria from 2014 were used to make the diagnoses (Rajkumar et al., 2014). Meanwhile, normal bone marrow samples from 14 healthy donors were collected as controls. Supplementary Table S1 depicts the distribution of clinical indicators and clinicopathological characteristics among the patients. The acquired samples were collected with the subjects’ informed consent. The study was authorized by the Ethics Committee of Wenzhou Medical University’s First Affiliated Hospital, and all operations were performed in accordance with the Helsinki Declaration.

### 2.9 Real-time fluorescence quantitative PCR

We validated the expression of five ferroptosis genes in MM using four human MM cell lines. Both the patients and the people in the control group had an aspirate of 5 mL taken from their bone marrow. The technique of density gradient centrifugation was utilized in order to isolate bone marrow mononuclear cells (BMMNC). RNA was extracted from the subjects ' BMMNC and myeloma cell lines using an RNA extraction kit (Yuanqi Bio, Shanghai, China) and reverse transcribed using HiScript ^®^ III RT SuperMix (Vazyme, Nanjing, China). The primers were purchased from Yimai Biotechnology Co. Ltd. β-actin was used as a relatively quantitative endogenous control. The relative mRNA expression of various molecules was calculated using the 2−δCt method. The Ct value of the molecules was then normalized to the β-Actin value. Each experiment was repeated three times. Then different test methods are used to compare the experimental results. The forward and reverse primers of each molecule are placed in Supplementary Table S2.

### 2.10 Statistical analyses

R 4.1.2 was used for the analysis of the data. Normalization and lognormality tests were also performed using the software known as Prism 9.0. Mann-Whitney and the *t*-test were used to compare two groups, while Kruskal-Wallis and ANOVA were utilized for the comparison of multiple groups.

## 3 Results

### 3.1 Baseline covariates and subject selection

The analysis included survival data from 1,512 subjects from three datasets. There were sufficient clinical covariates in GSE136337 (N = 417) and GSE24080 (N = 557) for univariate and multivariate Cox regression analysis, but not in GSE4204 (N = 538). The clinical covariates of all three datasets were converted into categorical variables, as shown in Table 1. 252 FRGs were extracted from the FerrDb database to determine the risk score of ferroptosis in the GSE136337 cohort. Finally, the GSE4204 and GSE24080 datasets are used to verify the model.

**TABLE 1:** Clinical messages of the training and validation cohorts.

Characteristics	Training cohort GSE136337 (N = 417)	Validation cohort GSE24080 (N = 557)	Validation cohort GSE4204 (N = 538)
Gender			
Female	158 (37.89%)	221 (39.68%)	
Male	259 (62.11%)	336 (60.32%)	
Age			
≤65	300 (71.94%)	430 (77.20%)	
>65	117 (28.06%)	127 (22.80%)	
Alb			
<3.5	85 (20.38%)	77 (13.82%)	
≥3.5	332 (79.62%)	480 (86.18%)	
β2M			
3.5–5.5	117 (28.06%)	120 (21.54%)	
<3.5	187 (44.84%)	318 (57.09%)	
>5.5	113 (27.10%)	119 (21.36%)	
LDH			
≤250	392 (94.00%)	507 (91.02%)	
>250	25 (6.00%)	50 (8.98%)	
ISS			
I	163 (39.09%)	287 (51.53%)	
II	134 (32.13%)	156 (28.01%)	
III	120 (28.78%)	114 (20.47%)	
R-ISS			
I	83 (19.90%)		
II	268 (64.27%)		
III	66 (15.83%)		
Survival			
Alive	239 (57.31%)	385 (69.12%)	445 (82.71%)
Dead	178 (42.69%)	172 (30.88%)	93 (17.29%)
Ferroptosis risk score			
High	208 (49.88%)	278 (49.91%)	269 (50.00%)
Low	209 (50.12%)	279 (50.09%)	269 (50.00%)
t (4,14)			
FALSE	403 (96.64%)		
TRUE	14 (3.36%)		
t (14,16)			
FALSE	416 (99.76%)		
TRUE	1 (0.24%)		
Treatment			
Transplants<=2	323 (77.46%)		
Transplants>2	94 (22.54%)		
TTII		344 (61.76%)	
TTIII		213 (38.24%)	

Alb albumin, β2M β2-microglobulin, LDH, lactate dehydrogenase. In validation cohort GSE24080, treatment included TT2 vs. TT3. TT2 and TT3 are clinical trials. TT2, UARK 98–026, Total Therapy II—a phase III study for newly diagnosed multiple myeloma evaluating anti-angiogenesis with thalidomide and post-transplant consolidation chemotherapy. TT3, UARK 2003–33, Total Therapy III—a phase 2 study incorporating bone marrow microenvironment (ME) co-targeting bortezomib into tandem melphalan-based auto-transplants with DT PACE for induction/consolidation and thalidomide + dexamethasone for maintenance.

### 3.2 The establishment of a prognostic ferroptosis risk score

**FIGURE 1:**
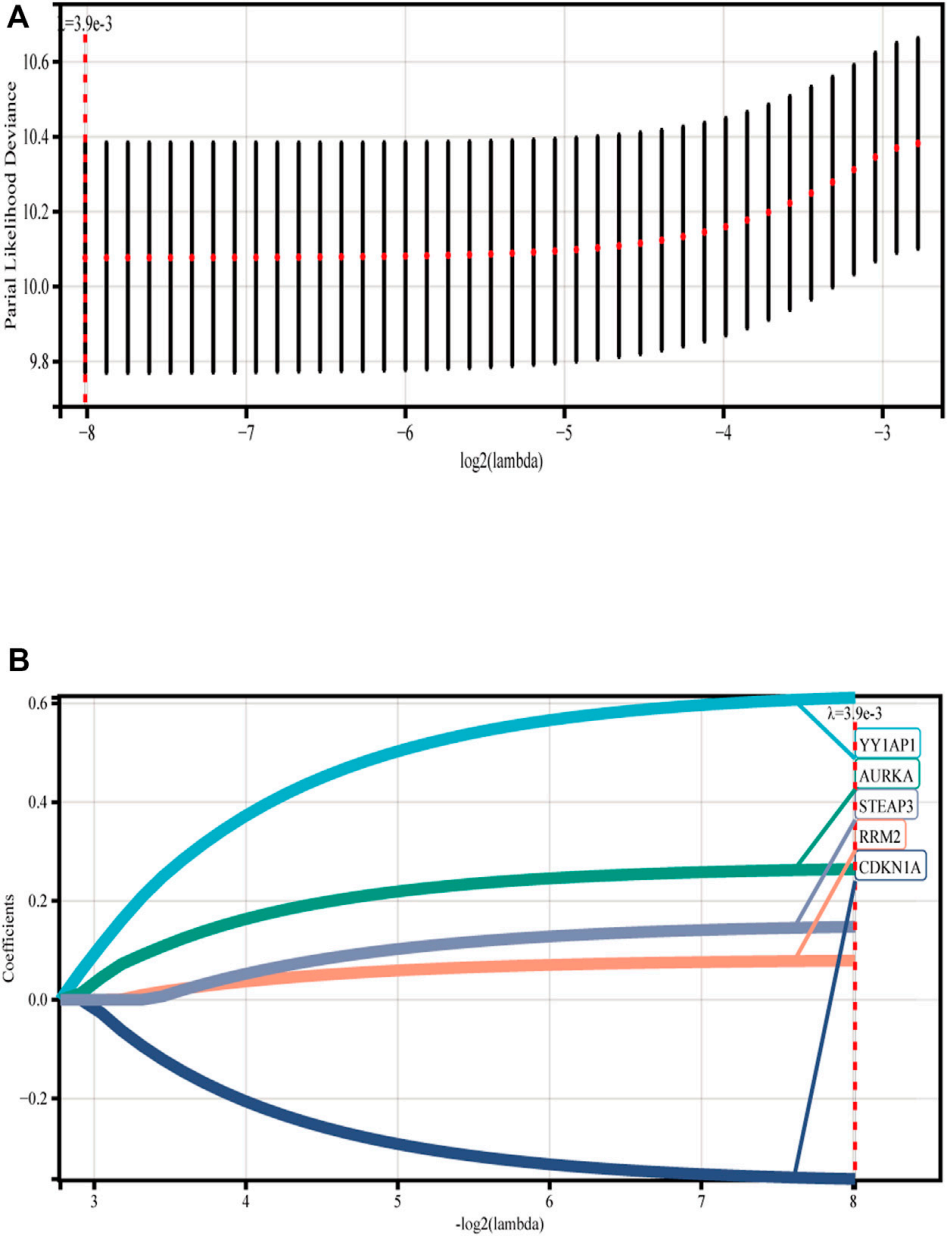
Construction of ferroptosis model. **(A)** Lasso Cox regression analysis was used for 10-fold cross-validation of variable selection. **(B)** LASSO coefficient of ferroptosis genes. Each curve represents a ferroptosis gene.

We discovered five FRGs that were significant predictors of survival (*p* < 0.001) using GSE136337 as the training data set for univariate Cox regression analysis (Supplementary Figure S1). The ferroptosis risk score was then generated by incorporating various data elements, such as survival status, gene expression, and the time of the survival. The Lambda value was 0.00387132108721305, and five genes were chosen (Figures 1A, B). Ferroptosis risk score = (0.611358×YY1AP1 expression) + (0.26426×AURKA expression) - (0.36283×CDKN1A expression) + (0.07901×RRM2 expression) + (0.14758×STEAP3 expression). The algorithm was then used to estimate each individual’s risk score, and based on the median value of the data set, it was used to divide each individual into low-risk and high-risk categories.

### 3.3 Validation of prognostic ferroptosis risk score in each cohort

**FIGURE 2:**
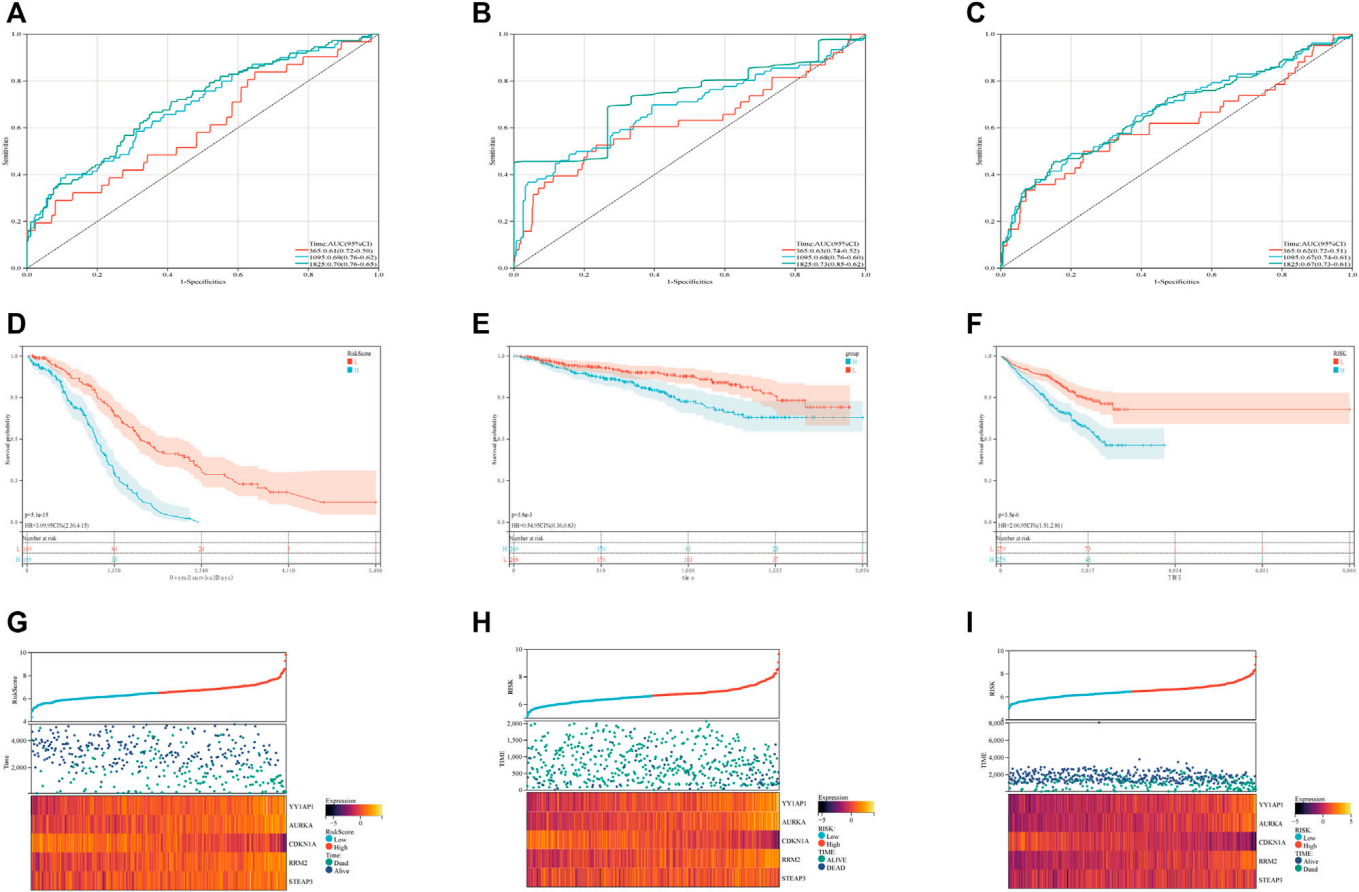
Validation of the ferroptosis risk-scoring model. **(A–C)** Sensitivity and specificity of the ferroptosis risk score model were assessed in each dataset by time-dependent ROC analysis. **(D–F)** Survival differences between high- and low-risk cohorts in each dataset. **(G–I)** Dot plots comparing outcomes of subjects in the high- and low-risk cohorts. The heat map displays results for the seven genes in both the training and validation cohorts. **(A)**, **(D)** and **(G)** display GSE136337; **(B)**, **(E)** and **(H)** display GSE4204; **(C)**, **(F)** and **(I)** display GSE24080.

In order to evaluate the levels of specificity and sensitivity exhibited by the ferroptosis risk scores, a time-ROC analysis was carried out. The AUCs of the 1, 3, and 5-year prognostic survival models based on FRGs expression to predict the survival of plasma cell myeloma in the GSE136337 training dataset were 0.61 (95% CI: 0.50, 0.72), 0.69 (0.62, 0.79), and 0.70 (0.65, 0.76), respectively (Figure 2A). Kaplan-Meier curves were used to compare the survival of high-risk and low-risk cohorts in the training set (Figure 2D) and validation sets GSE4204 and GSE24080 (Figures 2E, F). Compared with the low-risk cohort (61% [54, 68%] vs.84% [78, 88%]); 71% [64, 79%] vs. 87% [82, 92%]; 58% [52, 65%] vs. 75% [69, 81%]; subjects in the high-risk group had worse survival than those in the low-risk group. A dot plot was created to compare the survival rates of individuals in high-risk and low-risk populations, and it was found that in each data set (Figures 2G, I), low-risk populations had better survival than high-risk populations. We also compared the expression of these five FRGs using heat maps. Although the expression is slightly different in each data set, it is still relatively stable (Figures 2G, I).

### 3.4 Uni-variable and multi-variable analyses

We analyzed the gender, age, albumin, β2-microglobulin, LDH, t [4; 14], t [14; 16], del [17p], ISS and R-ISS by univariate Cox regression analysis. In the following multivariate COX regression analysis, albumin, β2-microglobulin and LDH were excluded due to their collinearity with ISS and R-ISS. The same analysis was also performed on the clinical covariates in the validation dataset (GSE24080) (Table 2). According to multivariate analysis, ferroptosis risk scores were independently associated with survival. The hazard ratio (HR) in the GSE136337 was 2.896 (95% CI: 2.250–3.728, *p* < 0.001), while the HR in the GSE24080 was 2.086 (95% CI: 1.623–2.681, *p* < 0.001).

**TABLE 2:** Univariate analysis and multi-variate regression analysis of overall survival in the training and validation cohorts by Cox regression analysis.

	Training cohort GSE136337(N = 417)				Validation cohort GSE24080 (N = 557)			
Characteristics	Uni-variate regression		Multi-variate regression		Uni-variate regression		Multi-variate regression	
	Hazard ratio (95%CI)	*p*-value	Hazard ratio (95%CI)	*p*-value	Hazard ratio (95%CI)	*p*-value	Hazard ratio (95%CI)	*p*-value
Gender		0.131				0.234		
Female	Reference				Reference			
Male	0.791 (0.586–1.069)	0.128			1.239 (0.876–1.751)	0.226		
Age		0.960				0.820		
≤65 years	Reference		Reference		Reference			
>65 years	1.009 (0.711–1.431)	0.960	1.774 (1.300–2.422)	<0.001	0.965 (0.712–1.309)	0.820		
Alb		0.839				<0.001		
≥3.5 g/dL	Reference				Reference			
<3.5 g/dL	0.956 (0.622–1.471)	0.839			2.878 (2.046–4.048)	<0.001		
B2M		0.529			1.613 (1.090–2.389)	0.017		
<3.5 mg/dL	Reference					<0.001		
≥5.5 mg/dL	0.938 (0.640–1.375)	0.743			Reference			
3.5–5.5 mg/dL	1.187 (0.833–1.691)	0.342			3.848 (2.627–5.638)	<0.001		
LDH		0.072				0.001		
≤250U/L	Reference				Reference			
>250U/L	0.568 (0.291–1.111)	0.099			1.917 (1.322–2.779)	<0.001		
ISS		<0.001				<0.001		
I	Reference		Reference		Reference		Reference	
III	2.739 (1.899–3.951)	<0.001	2.995 (1.745–5.142)	<0.001	1.600 (1.102–2.322)	0.013	1.456 (1.002–2.114)	0.049
II	1.533 (1.053–2.231)	0.026	1.328 (0.809–2.177)	0.262	3.067 (2.150–4.375)	<0.001	2.443 (1.700–3.509)	<0.001
Del (17p)		0.836						
FALSE	Reference							
TRUE	1.091 (0.482–2.469)	0.834						
t (4,14)		0.958						
FALSE	Reference							
TRUE	1.024 (0.420–2.499)	0.958						
t (14,16)		0.527						
FALSE	Reference							
TRUE	2.032 (0.284–14.527)	0.480						
Treatment numberoftransplants		0.212						
≤2	Reference							
>2	1.241 (0.889–1.732)	0.204						
TTII					Reference			
TTIII					0.798 (0.557–1.145)	0.221		
R-ISS		0.140						
I	Reference		Reference					
II	1.178 (0.807–1.720)	0.396	0.944 (0.542–1.642)	0.837				
III	1.653 (1.008–2.710)	0.047	0.580 (0.276–1.216)	0.149				
RiskScore	2.833 (2.220–3.616)	<0.001	2.896 (2.250–3.728)	<0.001	2.603 (2.056–3.296)	<0.001	2.332 (1.826–2.980)	<0.001

### 3.5 Creating a combined nomogram

**FIGURE 3:**
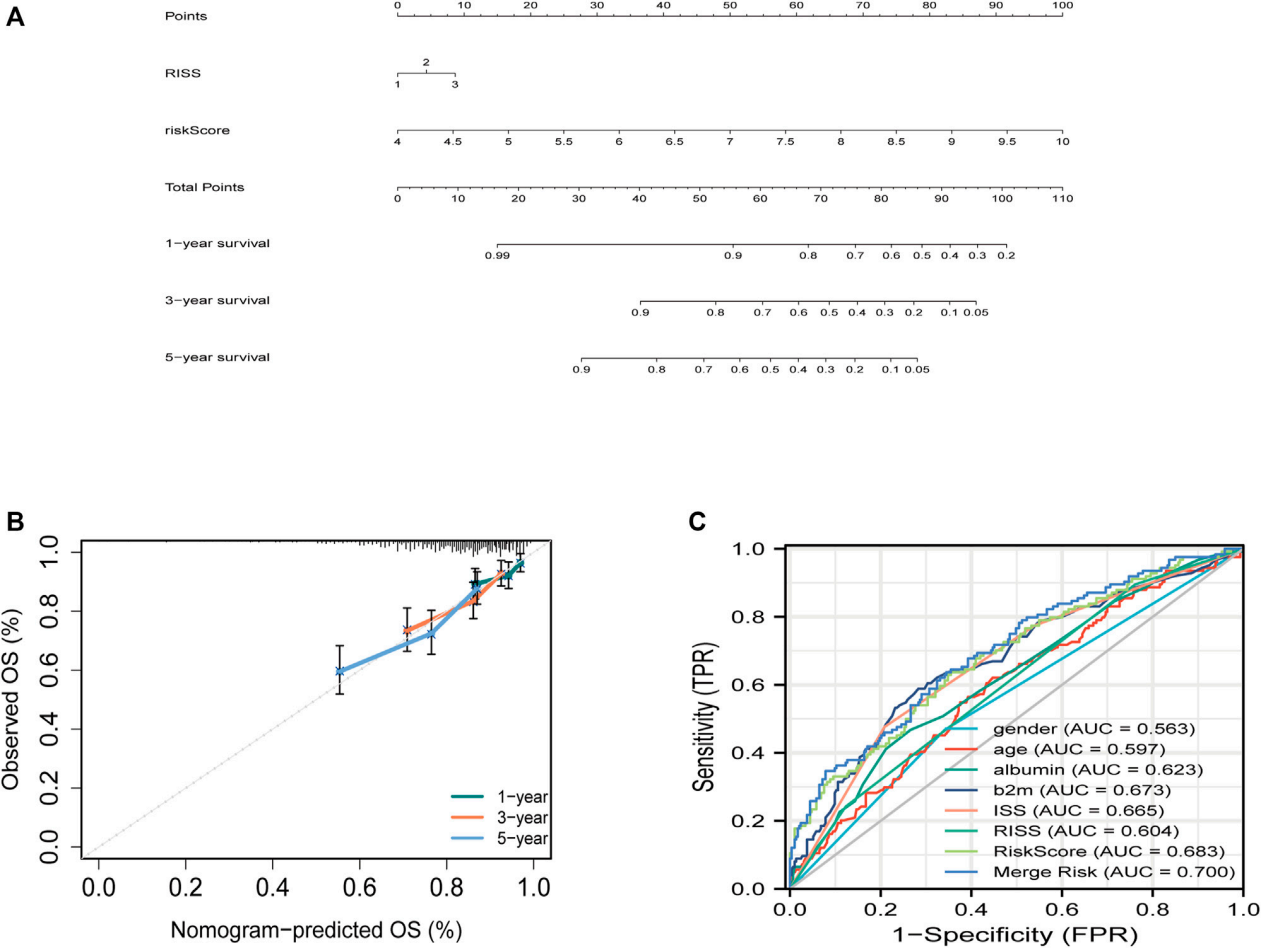
Building the combined nomogram to predict the overall survival (OS) of patients with multiple myeloma (MM). **(A)** The nomogram plot was built based on R-ISS staging, ferroptosis risk score and total points in the training cohort. **(B)** Timedependent receiver operating characteristic (ROC) curves of nomograms were compared based on co-variates 5-year survival. **(C)** Five-year ROC curves of the merged risk score compared with clinical covariates in the training dataset.

To predict survival, a joint nomogram model was constructed using R-ISS stage and disease-related ferroptosis risk score (Figure 3A). The calibration plot revealed that the nomogram was a great predictor of 1-, 3-, and 5-year survival (Figure 3B) The graph shows an increase in the prediction accuracy of 3-year survival rate and 5-year survival rate in the training cohort increased from 0.55 (0.49, 0.62) and 0.60 (0.56, 0.65) of R-ISS to 0.68 (0.61, 0.76) of AUC. *p* = 0.001), 0.70 (0.65, 0.76); *p* = 0.0016) (Figure 3C; Supplementary Figure S2). However, the validation data for the study did not have R-ISS. This means that the risk score could not be combined with the R-ISS. The 3- and 5-year AUCs calculated from the ISS for the GSE24080 dataset increased from 0.60 to 0.67 and 0.58 to 0.61, respectively (Supplementary Figure S3). In both the training and validation sets, the pooled risk score outperformed other factors like sex, age, albumin, and LDH.

### 3.6 Analyses of gene set enrichment

**FIGURE 4:**
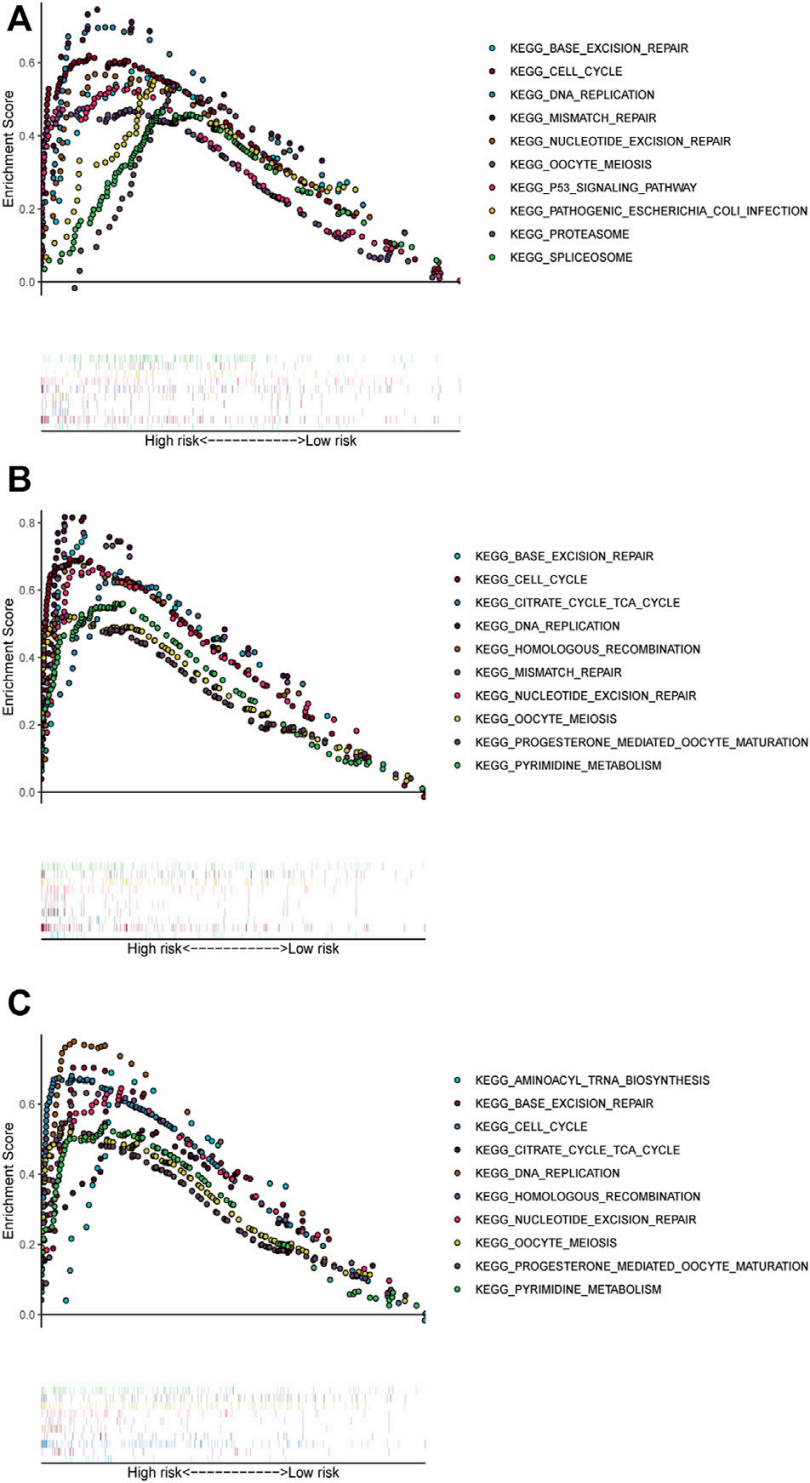
Pathways with significant enrichment in each dataset. The top 10 pathways were enriched in the training cohort **(A)** and validation datasets **(B–C)**.

We performed GSEA in all datasets, exploring ferroptosis-associated pathways and KEGG pathways associated with other ferroptosis covariates. Significantly enriched pathways, such as base excision repair and DNA replication, cell cycle, and mismatch repair, were concentrated in high-risk groups (Figure 4). Other ferroptosis-related pathways enriched in the high-risk group of the training and validation cohorts included mismatch repair, p53 signaling pathway, proteasome nucleotide excision repair, oocyte meiosis, pathogenic *Escherichia coli* infection, and spliceosome.

### 3.7 Drug sensitivity drug testing

**FIGURE 5:**
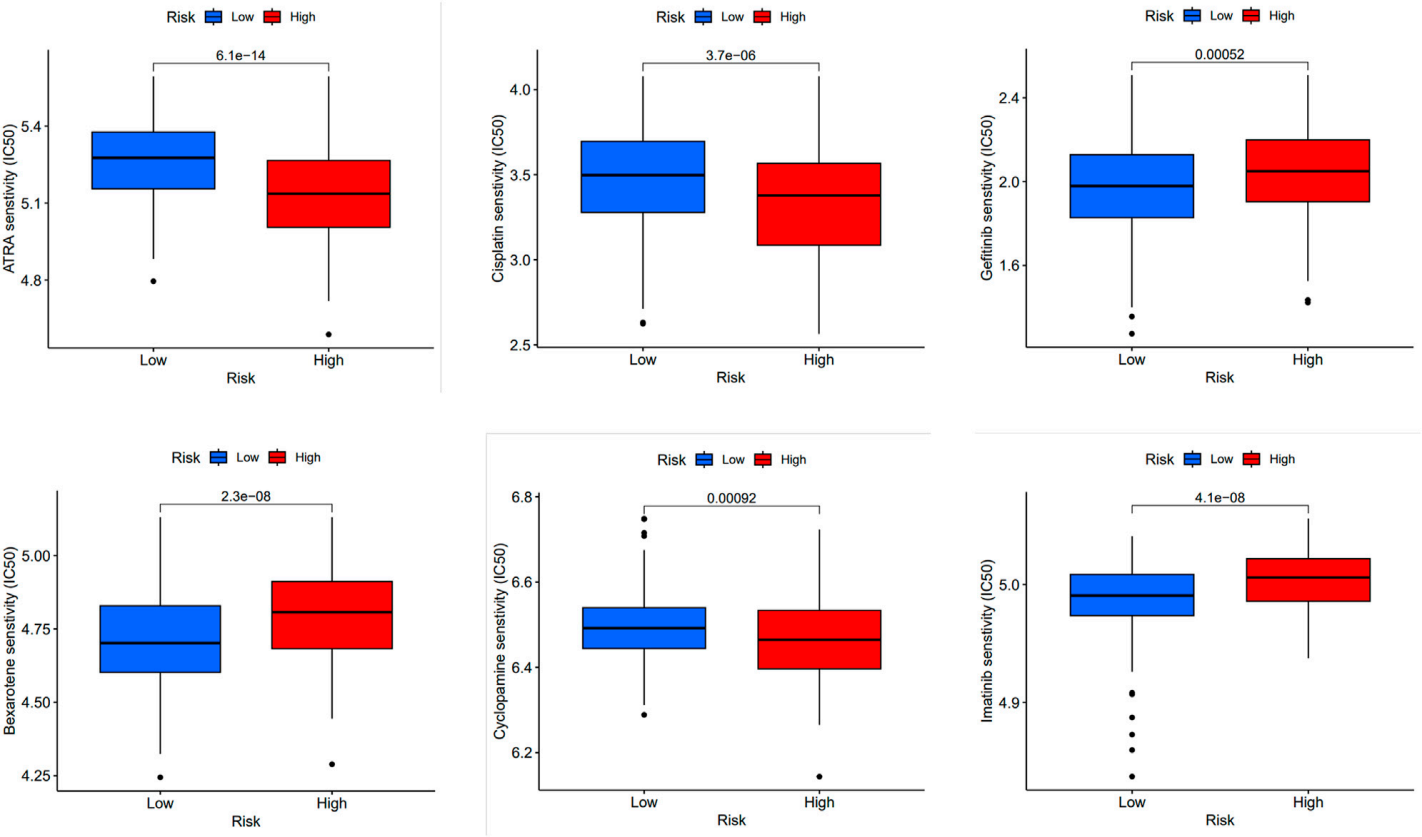
Prediction of drug sensitivity in high-risk group and low-risk group. Comparison of drug sensitivity between high-risk and low-risk groups was conducted. Statistical significance was determined at *p* < 0.05. The effectiveness of the drugs was measured using IC50 for the following drugs: ATRA, cisplatin, cyclopamine, bexarotene, gefitinib, and imatinib.

Furthermore, we compared the drug sensitivity of the high-risk with that of low-risk groups, and *p* < 0.05 was deemed statistically significant. The half inhibitory concentration (IC50), which symbolizes the concentration of the inhibitor needed to inhibit 50% of the drug, is used to measure the effectiveness of the drug. We found that all-trans-retinoic acid (ATRA), cisplatin, cyclopamine, bexarotene, gefitinib and imatinib had lower IC50 in high-risk groups, that is, higher sensitivity (Figure 5).

### 3.8 Immune-Related Analysis of MM Patients Using the Ferroptosis Score

**FIGURE 6:**
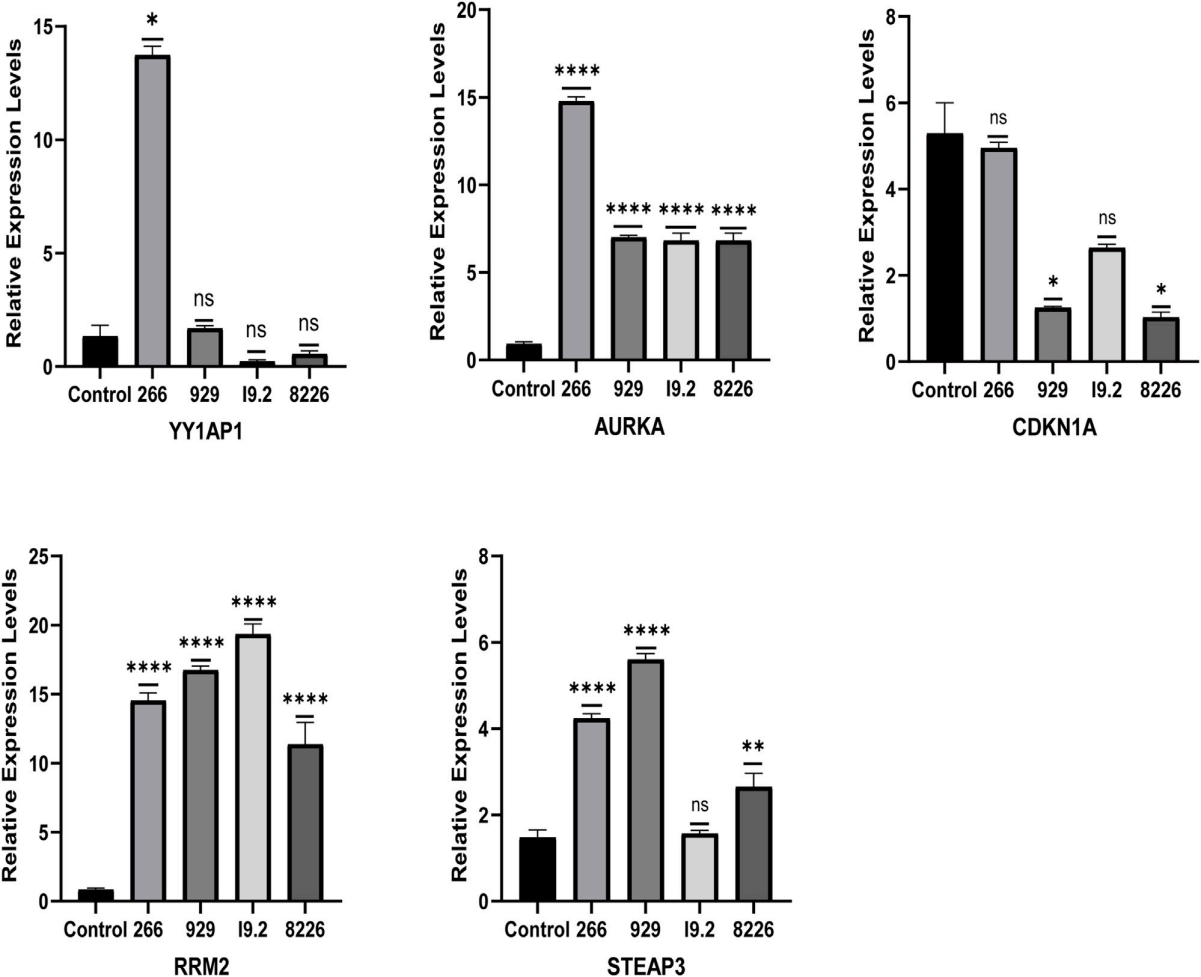
Expression of ferroptosis-related genes in normal human and MM cell lines. The relative mRNA expression levels of YY1AP1, AURKA, RRM2, STEAP3, and CDKN1A were analyzed using qRT-PCR in RPMI8226, H929, U266, and I9.2 cell lines.

To investigate the association between FRGs and antitumor immunity in MM patients, we utilized the CIBERSORT algorithm to evaluate immune cell infiltration in the GSE136337 dataset. The results revealed the proportional percentages of several immune cell types (Figure 6A). Stromal, immunological, and microenvironment scores were calculated to compare immune cell infiltration between individuals of different risk groups. Our analysis indicated that the scores of stromal, immune, and microenvironment were significantly higher in the high-risk group (*p* < 0.05) (Figure 6B). Furthermore, significant differences in activated CD4 memory T cells and Macrophage M0 were observed when comparing the proportions of different immune cell types in the two groups (Figure 6C). According to Pearson correlation analysis, the expression levels of FRGs showed a significant correlation with immune cell populations (*p* < 0.05). Within the risk model, three FRGs were found to be associated with T cell CD4 memory activation. Specifically, CDKN1A, the only protective gene in the model, exhibited a negative correlation with T cells CD4 memory activated, while AURKA and RRM2 showed a positive correlation (Figure 6D). Additionally, we investigated the relationship between FRGs and the tumor immune dysfunction and exclusion (TIDE) score. Our findings revealed significant correlations between AURKA, CDKN1A, and STEAP3 and tumor exclusion (Figure 6E). We also evaluated the immune prognostic signature (IPS) scores in two groups and found higher IPS scores in the high-risk group, indicating greater responsiveness to immunotherapy (Figure 6F).

### 3.9 Relative mRNA expression levels of cell lines and patients

**FIGURE 7:**
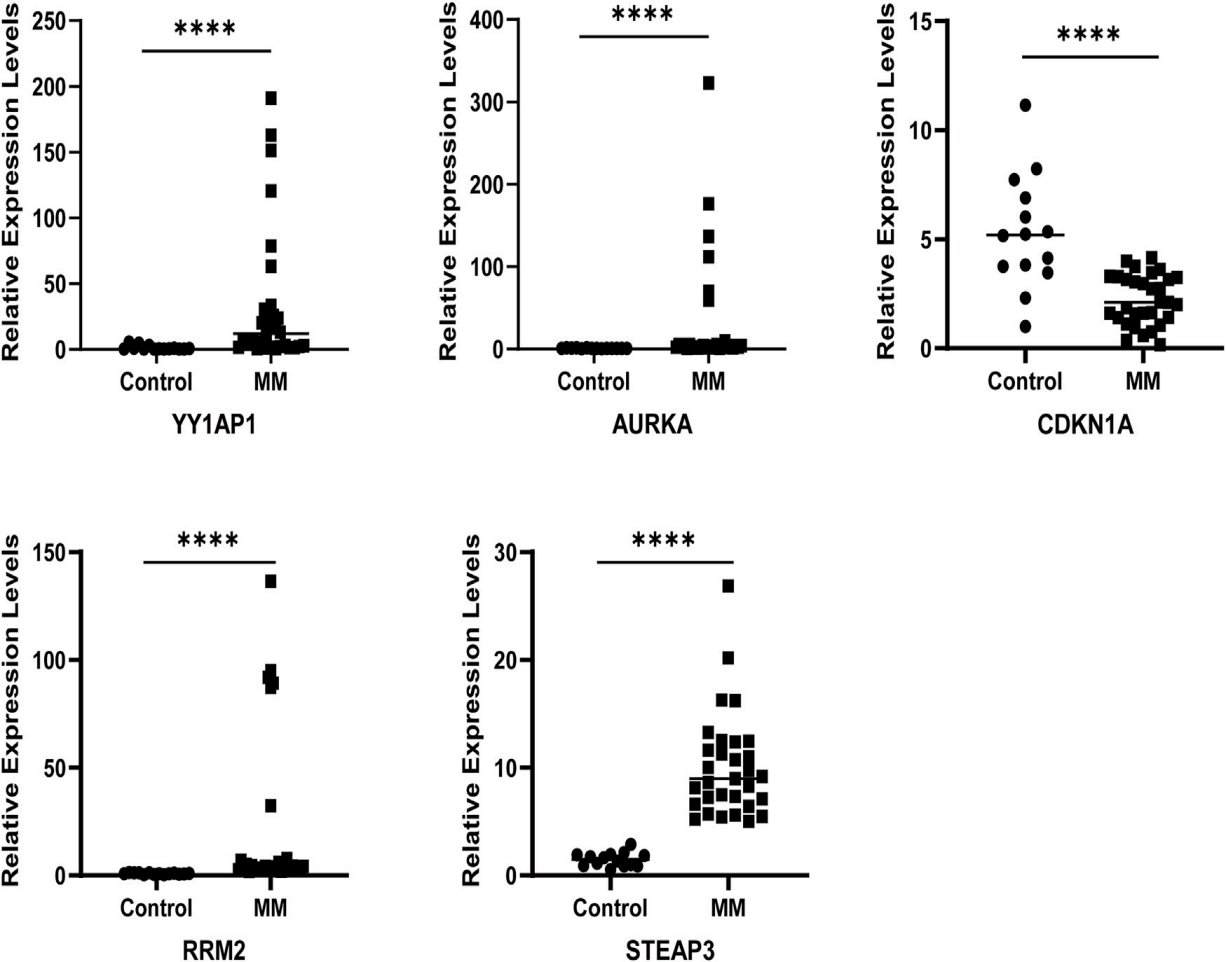
Expression of ferroptosis-related genes in normal human and MM patients. The relative mRNA expression levels of YY1AP1, AURKA, RRM2, STEAP3, and CDKN1A were analyzed using qRT-PCR in BMMNC derived from 31 MM patients.

**FIGURE 8:**
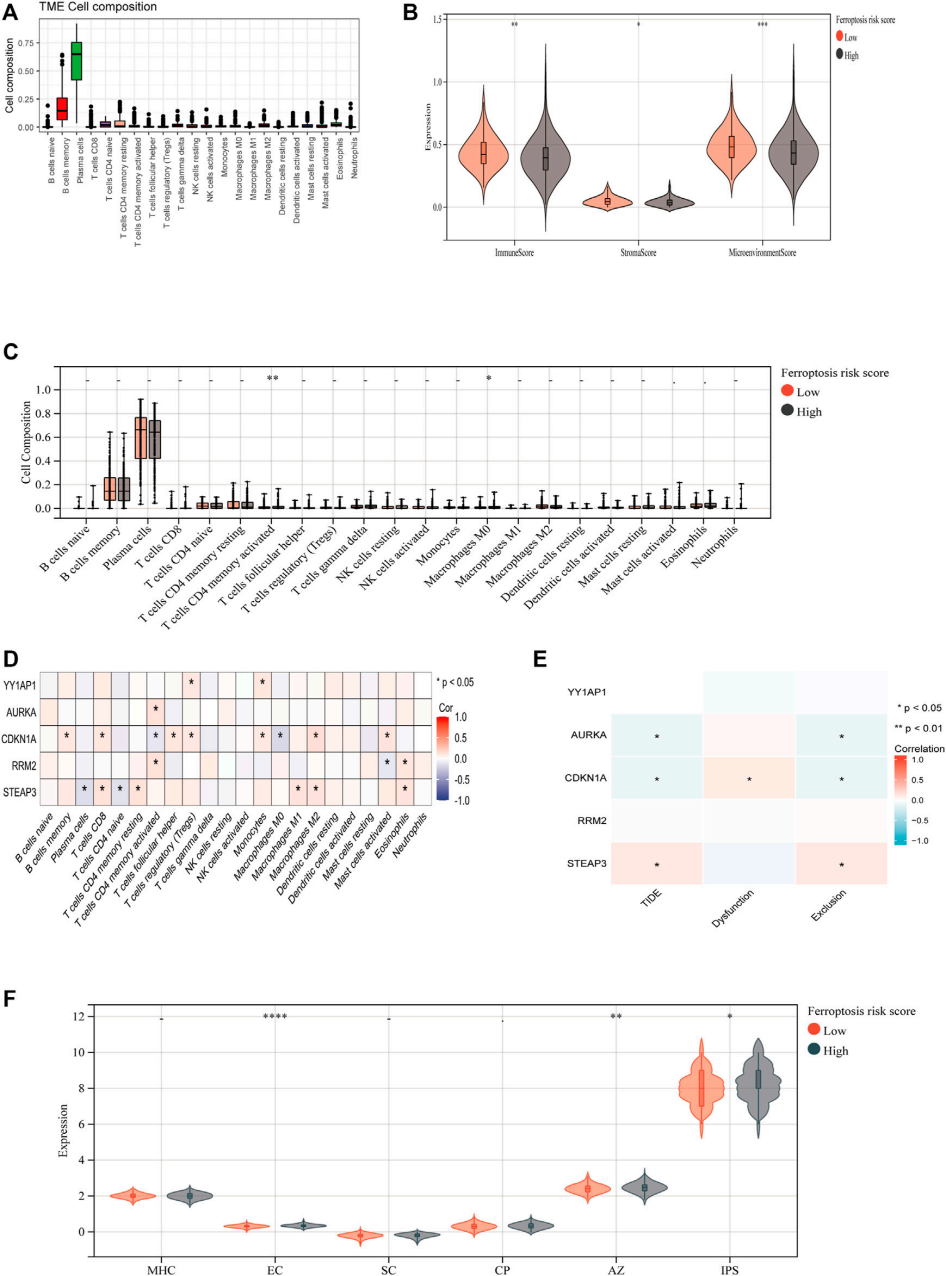
Immune-Related Analysis of MM Patients Using the Ferroptosis Score. **(A)** CIBERSORT algorithm determined the immune cell infiltration of all MM patients with GSE136337, showing the proportion of each typical immune cell. **(B) **The XCELL algorithm compared the matrix score, immune score and microenvironment score between the high-risk group and the low-risk group. **(C)** Compare the proportion of each immune cell between high-risk and low-risk groups. **(D)** The correlation between ferroptosis-related genes and immune cells was analyzed by Spearman statistical method. **(E)** Correlation analysis between model ferroptosis genes and TIDE score. **(F)** IPS model scores of high-risk group and low-risk group.

The qRT-PCR results showed that the relative mRNA expression for YY1AP1, AURKA, RRM2, and STEAP3 in RPMI8226, H929, U266 and I9.2 was statistically significantly higher than the control, while CDKN1A was decreased (Figure 7). In addition, compared with the control, the relative mRNA expression of YY1AP1, AURKA, RRM2, STEA3 were significantly increased in BMMNC of 31 MM patients while the expression of CDKN1A was decreased, which is consistent with our previous analysis results (Figure 8).

## 4 Discussion

We developed a new prognostic risk score for ferroptosis based on 5-FRGs, using the GSE136337 dataset as the training dataset. We validated this score in two independent datasets, and in multivariate analysis with R-ISS, the ferroptosis risk score was confirmed as an independent prognostic factor.

Our ferroptosis risk score identified CDKN1A as a positive prognostic factor and YY1AP1, AURKA, RRM2, and STEAP3 as negative prognostic factors. AURKA, a serine/threonine kinase that regulates cell division during mitosis, was overexpressed in several tumor types compared to normal tissues according to TCGA database. It has been shown to promote the development of hematological malignancies and solid tumors (Du et al., 2021).

RRM2, a stress response factor, has been associated with the initiation and advancement of various cancers. Analysis of the Oncomine database showed elevated expression of RRM2 in MM patients in comparison to healthy controls. In addition, downregulation of RRM2 by targeting the Wnt/-catenin signaling pathway may promote MM cell death and activate DNA damage (Liu et al., 2019).

CDKN1A is responsible for encoding P21, which is a founding member of the cyclin-dependent kinase inhibitor family (CKI) (Xiong et al., 1993). CKI is a crucial regulator of the cell cycle that maintains genomic stability, and is frequently dysregulated in human cancers (Roninson, 2002; Abbas and Dutta, 2009; Kreis et al., 2015). Studies have shown that downregulation of p21 expression can promote the proliferation of MM cells (Xiang et al., 2021). Highly expressed CDKN1A may play a tumor suppressor role by encoding p21.

Prostate family member 3 (STEAP3) six transmembrane epithelial antigen was originally discovered on mouse M1 bone marrow cells. According to research, STEAP3 plays a critical role in the regulation of ferroptosis via modulating iron metabolism (Mou et al., 2019). The proliferation of cancer cells, including, pancreatic cancer (Yu et al., 2020), colorectal cancer (Barresi et al., 2016) and bladder cancer (Kim et al., 2016) may be facilitated by the overexpression of STEAP3, which promotes iron uptake and storage.

Based on the results of GSEA, the high-risk score group demonstrated a significant enrichment of ferroptosis-related pathways. Among them, the base excision repair (BER) pathway was notably enriched, which may suggest that cancer cells depend on heightened BER activity to withstand oxidative stress. The proteasome pathway was also enriched, which is a myeloma cell-dependent proteasome complex that manages protein overload by collecting misfolded proteins in the endoplasmic reticulum. Additionally, DNA replication-enriched pathways were found to promote cell biosynthesis.

We performed a drug sensitivity test with high and low risk groups. ATRA is a vitamin A derivative. The anti-IL-6 drug ATRA can inhibit the growth of human MM cell lines. It has also been shown that the combination of this drug and the other medication, daratumumab, can effectively treat patients with this type of cancer (Frerichs et al., 2021). The ATRA works by increasing the CD38 expression in cancer cells and boosting the complement-dependent and antibody-mediated cytotoxic properties of daratumumab. It also increased the activity of the drug in a mouse model of MM (Nijhof et al., 2015). After large-scale data analysis of the gene spectrum of MM patients, it can be confirmed that ATRA can make human myeloma cells sensitive to carfilzomib, a proteasome inhibitor, by activating the retinoic acid receptor (RAR) γ and interferon-β response pathways, thereby effectively improving their survival (Wang et al., 2022). Cyclopamine has the ability to make myeloma cells more susceptible to apoptosis by circulating replacement TRAIL (Wang et al., 2021). Imatinib mesylate (STI571) has the ability to limit the proliferation of multiple myeloma (MM) cells and boost the effectiveness of anti-myeloma medicines like dexamethasone, which are routinely utilized (Pandiella et al., 2003). Understanding the drug sensitivity genomics of MM can better arrange targeted anti-myeloma treatment, which is also of great significance for myeloma drugs such as bortezomib (Ghasemi et al., 2016).

Using the XCELL algorithm, the data collected by the study revealed that the immune infiltration levels of high-risk individuals were more significant than those of the lower-risk group. It suggested that treating them with immunotherapies could improve their cancer responses. Using the XCELL algorithm, the data collected by the study revealed that the immune infiltration levels of high-risk individuals were more significant than those of the lower-risk group. It suggested that treating them with immunotherapies could improve their cancer responses (MacLeod et al., 2010). These mechanisms can be utilized by tumor-specific CD4 + T cells to deliver effective anti-cancer immune responses. Some of these include the licensing of CD8 + T cells (Schietinger et al., 2010), cytotoxic killing of tumor cells expressing MHC-II (Lundin et al., 2003; Quezada et al., 2010), macrophage (Corthay et al., 2005) and natural killer (NK) cell activation (Corthay et al., 2005) and cytokine-mediated effects on the tumor vascular system (Qin and Blankenstein, 2000). And the correlation analysis of FRGs with TIDE score and T cell exclusion and dysfunction score was significant. It is possible that these factors are responsible for the higher immunological score exhibited by the high-risk group. And the IPS model score also fully proves this finding. The MM ferroptosis risk score can help predict the disease’s prognosis and play a crucial role in the development of immunotherapies for this condition.

Our study is not without limitations. Firstly, some potential prognostic factors could not be accounted for in all three datasets due to missing information, with validation cohorts only including ISS data. Secondly, treatment effects were not adjusted for in the data, making our scores more prognostic than predictive. Thirdly, it is not known how much each gene contributes individually to the ferroptosis score, which is something that has to be investigated further. Lastly, the specific role of YY1AP1 in MM remains unclear. Future studies should focus on adjusting for treatment effects to enhance the predictive capabilities of the scores and improve patient outcome assessment. Additionally, investigating the function and significance of YY1AP1 in multiple myeloma, including its interactions with other genes and pathways, can provide valuable insights for disease progression and treatment strategies. Moreover, integrating phenomics into the research can also deepen our understanding of how genes, epigenetics, symbiotic microorganisms, diet, and environmental exposure contribute to multiple myeloma, potentially leading to the discovery of novel biomarkers and therapeutic targets (Liu et al., 2022; Ying, 2023).

In conclusion, we have created and validated a prognostic model for MM that is based on the expression of FRGs in MM cells. Our ferroptosis risk score proved to be a distinct predictor of survival after subjecting it to multivariate analysis. Individuals with high-risk scores can also have better options in the selection of chemotherapy drugs. And ferroptosis risk score may play an important role in the guidance of immunotherapy in clinical MM patients. *In vitro* experiments confirmed the expression of FRGs in MM patients. The addition of our ferroptosis risk score to R-ISS improved the accuracy of survival prediction and could potentially be used in combination with R-ISS for prognostic purposes.

## Data Availability

The datasets presented in this study can be found in online repositories. The names of the repository/repositories and accession number(s) can be found in the article/Supplementary Material.
