# Supplementary material for: Comprehensive analysis of ferroptosis-related genes in immune infiltration and prognosis in multiple myeloma
Source: Front Pharmacol. 2023 Aug 7;14:1203125. doi: 10.3389/fphar.2023.1203125 (PMC10440437; doi:10.3389/fphar.2023.1203125)

**Supplementary Table 1**

|  |  |  |  |  |  |  |  |  |  |  |  |  |  |  |  |  |  |
| --- | --- | --- | --- | --- | --- | --- | --- | --- | --- | --- | --- | --- | --- | --- | --- | --- | --- |
| ID | Sex | Age | Isotype | Albumin | β2m | LDH | Del (17p) | Del (13q) | Amp1q | IgH rearrangement | ISS | R-ISS | Myeloma cells | Calcium | Serum creatinine | Hb | Bone lesions |
| 1 | 1 | 61 | IgA | 3.23 | 1.369 | 145 | FALSE | FALSE | FALSE | FALSE | 2 | 2 | 8.5 | 2.3 | 87 | 99 | 2 |
| 2 | 2 | 65 | IgA | 4.36 | 1.292 | 147 | FALSE | TRUE | FALSE | FALSE | 1 | 2 | 8 | 2.34 | 45 | 120 | 2 |
| 3 | 1 | 59 | IgG | 3.26 | 4.071 | 932 | FALSE | FALSE | FALSE | FALSE | 2 | 2 | 40 | 2.44 | 153 | 117 | 2 |
| 4 | 1 | 66 | IgG | 2.91 | 4.019 | 189 | FALSE | TRUE | TRUE | FALSE | 2 | 2 | 18.5 | 2.01 | 90 | 117 | 0 |
| 5 | 1 | 86 | IgG | 3.07 | 3.392 | 215 | FALSE | TRUE | TRUE | FALSE | 2 | 2 | 17.5 | 2.05 | 80 | 75 | 0 |
| 6 | 1 | 49 | light | 4.35 | 2.126 | 212 | FALSE | FALSE | TRUE | FALSE | 1 | 2 | 29.5 | 3.21 | 91 | 143 | 2 |
| 7 | 2 | 68 | IgG | 3.57 | 5.625 | 169 | FALSE | TRUE | FALSE | FALSE | 3 | 2 | 13.5 | 2.45 | 98 | 87 | 2 |
| 8 | 2 | 55 | IgA | 3.75 | 8.932 | 132 | FALSE | TRUE | TRUE | FALSE | 3 | 2 | 51.5 | 2.24 | 43 | 80 | 2 |
| 9 | 1 | 73 | light | 3.35 | 3.822 | 268 | FALSE | FALSE | FALSE | FALSE | 2 | 2 | 0.5 | 1.89 | 130 | 88 | 2 |
| 10 | 1 | 75 | IgG | 2.41 | 7.451 | 125 | FALSE | FALSE | TRUE | FALSE | 3 | 2 | 17.5 | 1.97 | 130 | 62 | 0 |
| 11 | 1 | 69 | IgG | 3.32 | 2.875 | 224 | FALSE | FALSE | FALSE | FALSE | 2 | 2 | 0.5 | 2.07 | 97 | 127 | 0 |
| 12 | 1 | 59 | IgG | 2.89 | 6.879 | 204 | FALSE | FALSE | FALSE | FALSE | 3 | 2 | 59 | 2.22 | 83 | 87 | 2 |
| 13 | 1 | 67 | IgA | 3.23 | 11.908 | 146 | FALSE | FALSE | FALSE | FALSE | 3 | 2 | 3.5 | 2.34 | 208 | 52 | 2 |
| 14 | 1 | 76 | IgG | 3.2 | 33.153 | 254 | FALSE | TRUE | TRUE | TRUE | 3 | 2 | 37 | 2.14 | 382 | 82 | 0 |
| 15 | 1 | 79 | IgA | 3.75 | 9.323 | 142 | FALSE | FALSE | FALSE | FALSE | 3 | 2 | 15 | 2.24 | 253 | 64 | 0 |
| 16 | 2 | 74 | IgA | 3.27 | 2.427 | 111 | FALSE | FALSE | FALSE | FALSE | 2 | 2 | 10 | 2.36 | 54 | 111 | 1 |
| 17 | 2 | 69 | IgG | 3.54 | 2.428 | 149 | FALSE | FALSE | FALSE | FALSE | 1 | 2 | 26.5 | 2.24 | 60 | 107 | 2 |
| 18 | 1 | 69 | light | 4.03 | 31.861 | 292 | FALSE | FALSE | FALSE | FALSE | 3 | 2 | 14.5 | 2.37 | 574 | 71 | 0 |
| 19 | 2 | 77 | IgG | 3.34 | 2.132 | 155 | FALSE | FALSE | FALSE | FALSE | 2 | 2 | 20 | 2.17 | 49 | 60 | 0 |
| 20 | 1 | 67 | IgA | 2.74 | 2.623 | 263 | FALSE | FALSE | FALSE | FALSE | 2 | 2 | 16 | 2.62 | 78 | 96 | 2 |
| 21 | 1 | 65 | light | 4.36 | 9.88 | 176 | FALSE | FALSE | FALSE | FALSE | 3 | 2 | 8.5 | 2.41 | 462 | 72 | 2 |
| 22 | 2 | 70 | IgG | 3.29 | 2.49 | 157 | FALSE | FALSE | FALSE | FALSE | 2 | 2 | 8 | 2.05 | 86 | 105 | 0 |
| 23 | 2 | 55 | IgD | 3.8 | 1.566 | 176 | FALSE | FALSE | FALSE | FALSE | 1 | 2 | 1 | 2.31 | 35 | 121 | 2 |
| 24 | 2 | 78 | IgG | 2.86 | 4.157 | 787 | FALSE | FALSE | TRUE | FALSE | 2 | 2 | 89 | 2.38 | 43 | 79 | 2 |
| 25 | 2 | 53 | IgG | 2.85 | 1.869 | 457 | FALSE | TRUE | FALSE | FALSE | 2 | 2 | 2 | 2.29 | 71 | 92 | 2 |
| 26 | 1 | 55 | IgA | 2.49 | 4.455 | 75 | FALSE | FALSE | FALSE | FALSE | 2 | 2 | 13 | 2.14 | 93 | 86 | 1 |
| 27 | 1 | 75 | IgG | 3.73 | 7.764 | 173 | FALSE | TRUE | TRUE | FALSE | 3 | 2 | 17.5 | 2.32 | 201 | 85 | 0 |
| 28 | 1 | 62 | IgG | 2.91 | 6.542 | 126 | FALSE | FALSE | TRUE | FALSE | 3 | 2 | 28.5 | 2.29 | 124 | 113 | 2 |
| 29 | 1 | 80 | IgG | 2.23 | 4.952 | 184 | FALSE | TRUE | FALSE | FALSE | 2 | 2 | 38.5 | 2.03 | 73 | 91 | 2 |
| 30 | 1 | 65 | light | 4.18 | 10.486 | 178 | FALSE | FALSE | FALSE | FALSE | 3 | 2 | 4 | 2.39 | 390 | 71 | 2 |
| 31 | 2 | 80 | light | 3.69 | 5.902 | 198 | FALSE | FALSE | TRUE | FALSE | 3 | 2 | 42 | 2.27 | 84 | 79 | 0 |

**Supplementary Table 2**

| **YY1AP1** | **Forward-5′AACCCAGGGAGTCGGCTTA 3′** |
| --- | --- |
|  | **Reverse-5′CGATGAGAGTACAGGGAAGTGA 3′** |
| **AURKA** | **Forward-5′GGAATATGCACCACTTGGAACA 3′** |
|  | **Reverse-5′TAAGACAGGGCATTTGCCAAT 3′** |
| **CDKN1A** | **Forward-5′TGTCCGTCAGAACCCATGC 3′** |
|  | **Reverse-5′AAAGTCGAAGTTCCATCGCTC 3′** |
| **RRM2** | **Forward-5′CACGGAGCCGAAAACTAAAGC 3′** |
|  | **Reverse-5′TCTGCCTTCTTATACATCTGCCA 3′** |
| **STEAP3** | **Forward-5′GTGGACAGCGATAGTAGCCTTGC 3′** |
|  | **Reverse-5′TGTGCGTTTGGGGTTGCG 3′** |

**Supplementary Fig S1**


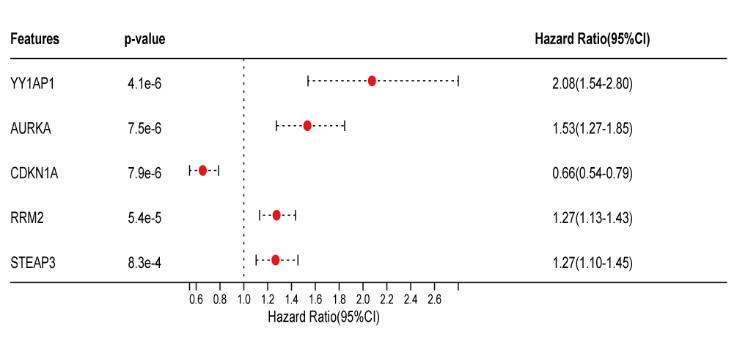


**Fig S2 Three-year ROC curves of the merged risk score compared with clinical covariates in the training dataset.**


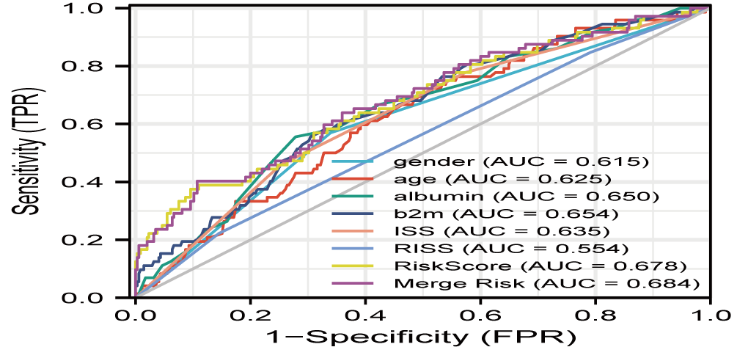


**FigS3**

**(A)Three-year ROC curves of the merged risk score compared with clinical covariates in the training dataset.**

**(B)Five-year ROC curves of the merged risk score compared with clinical covariates in the training dataset.**


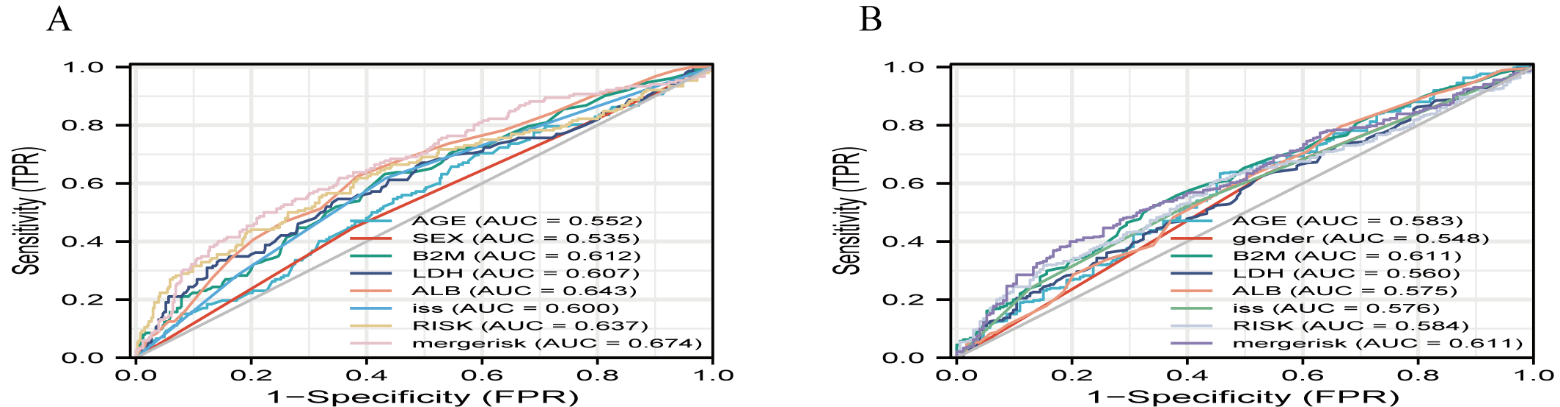


**Fig S4 The experiment of FeAc on cells in vitro**


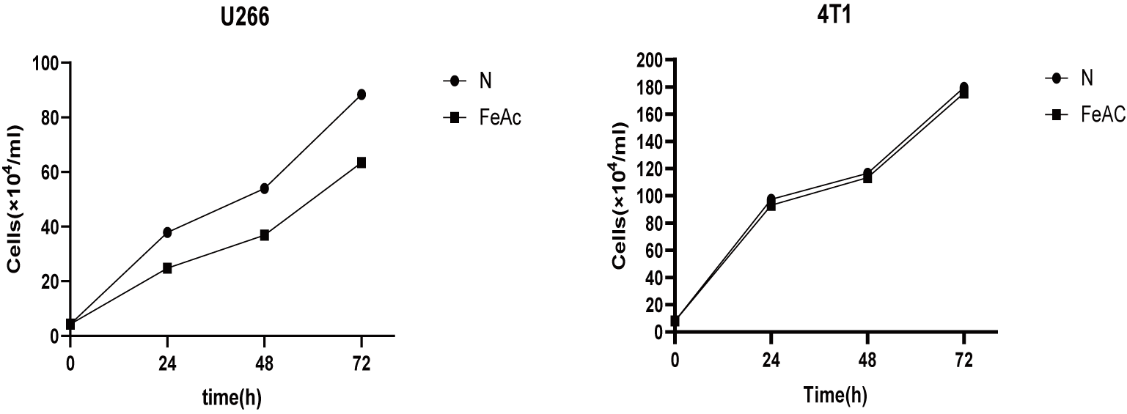

Supplement: Supplementary file 1 [file DataSheet1.docx]
